# Ifosfamide-induced encephalopathy: the EEG with frontal intermittent delta activity, and rapid resolution with methylene blue: A case report

**DOI:** 10.1186/s13569-020-00147-3

**Published:** 2020-11-28

**Authors:** Juliette E. Hamilton, Michael Alexander, Fergal C. Kelleher

**Affiliations:** 1 Department of Medical Oncology, Trinity College Dublin, St James’s Hospital, Dublin 8, Ireland; 2 Department of Neurophysiology, Tallaght University Hospital, Dublin, Ireland

**Keywords:** Ifosfamide, Encephalopathy, Methylene blue, Frontal rhythmic intermittent delta activity FIRDA

## Abstract

**Background:**

Encephalopathy is an established side effect of the chemotherapeutic agent, ifosfamide, occurring in 10–30% of cases. The EEG commonly shows non-specific features of encephalopathy, and rarely shows frontal intermittent rhythmic delta activity (FIRDA).

**Case presentation:**

This is a case report of a 71 year old woman with pleomorphic sarcoma, who developed ifosfamide-induced encephalopathy with her second dose of ifosfamide. It shows the characteristic EEG findings that have been described previously with ifosfamide-induced encephalopathy and additionally the unusual and rare finding of FIRDA. This was followed up by a further EEG showing resolution of the encephalopathy, after administration of methylene blue, coinciding with rapid and complete resolution of her symptoms.

**Conclusion:**

The rapid resolution of the encephalopathy on the EEG after administration of methylene blue adds further evidence to its effectiveness as a treatment for the disorder.

## Background

Ifosfamide (3-[2-chloroethyl]-2-[(2-chloroethyl)-amino] tetrahydro-2H-1, 3,2-oxazaphosphorin-2-oxide) is a chemotherapeutic agent used for a variety of solid organ and haematological malignancies, including in the management of sarcomas [1, 2]. It is a prodrug that is metabolized in the liver into the active alkylating agents by cytochrome P450 enzymes [3]. The alkylating agents produced are 4-hydroxy-ifosfamide and ifosfamide mustard with other resultant products including chloroacetaldehyde and chlorothylamine. Chloroacetaldehyde and chlorothylamine are thought likely to be the major contributing factors in the development of encephalopathy, as they are known to be neurotoxic and are able to penetrate the blood–brain barrier [3, 4]. These metabolites are excreted predominantly by the kidneys [2].

Neurotoxicity can manifest in several ways, including lethargy, agitation, disorientation, confusion, hallucinations, extra-pyramidal signs and seizures [1, 5]. In rare cases, the symptoms can progress to coma, irreversible brain damage and death [1]. These symptoms are thought to develop due to glutaric acid accumulation, which may inhibit mitochondrial respiration [4]. This specific pathway was identified after glutaric acid was found in the urine of patients with congenital glutaric aciduria, a congenital metabolic disorder which results in a lack of the flavoproteins for electron transfer in mitochondrial respiration [6]. This disturbance leads to an increase in intracellular nicotinamide adenine dinucleotide, inhibiting the dehydrogenation and oxidation of chloroacetaldehyde and chlorothylamine [3].

There are many hypothesised risk factors for the development of ifosfamide-induced encephalopathy. Advanced age, poor performance status, impaired renal and liver function, past history of intracerebral pathology, platinum exposure, malnutrition evidenced by low albumin, higher levels of haemoglobin, concomitant use of aprepitant and lower gastrointestinal and pelvic malignancies [3, 7]. Aprepitant is a CYP 3A4 enzyme inhibitor, and therefore has the potential to increase the levels of chloroacetaldehyde and chlorothylamine [1].

Ifosfamide-induced encephalopathy is a clinical diagnosis, with onset within 2 h up to 146 h after the commencement of the ifosfamide infusion [1]. It is graded by severity, with grade 1 encompassing a vague or slightly depressive affect, grade 2 resulting in extensive periods of sleep, restlessness or agitation, grade 3 manifests as stupor, heavy depression or mild hallucinations and grade 4 expressing overt hallucinations, seizures or coma [2, 3].

Resolution of symptoms have been observed with the administration of methylene blue. Whilst the exact mechanism of action is unclear, it is felt that it serves as an alternative electron acceptor. This prevents chlorothylamine from inhibiting flavoprotein and so corrects mitochondrial respiration [1]. It also inhibits extra-hepatic monoamine oxidases, preventing the dehydrogenation of aldehydes, including the formation of chloroacetaldehyde [1, 2, 6]. The toxicities of methylene blue include cardiac arrhythmias, coronary vasoconstriction, syncope, hemolytic anemia and anaphylaxis [8].

Thiamine has also been used in the management of ifosfamide-induced encephalopathy, specifically because of the similar clinical syndrome of Wernicke’s encephalopathy, and has been found to be effective [2]. Additionally, albumin administration to provide binding sites that are unable to cross the blood brain barrier, and haemodialysis, have been found to be effective [2].

## Case presentation

Our patient was a 71 year old woman diagnosed with a high-grade right triceps pleomorphic sarcoma in 2017, which was resected with a wide local excision and treated with adjuvant radiation. A surveillance computed tomography (CT) scan of the thorax showed metastases to the lung and mediastinum in September 2018 and she was treated with 6 cycles of doxorubicin. Progression of disease identified in January 2019 necessitated a change of treatment to ifosfamide and mesna, and she was admitted for each cycle. Re-staging CT scan after 2 cycles demonstrated an interval decrease in the size and number of the multiple pulmonary nodules with one nodule reducing in size from 18 mm down to 6 mm, complete resolution of a 15 mm right para-oesophageal node and no new disease identified.

Past medical history included hypertension, hysterectomy for menorrhagia and appendectomy. Her medications on admission were lercanidipine, losartan, hydrochlorothiazide, potassium supplement, enoxaparin (thrombosis prophylaxis), senna, domperidone, aprepitant, Fortisip Compact Fibre, Procal Shot, multivitamin and evening primrose oil (not administered due to unknown possible drug interactions). She was known to develop a rash with penicillin but had no other allergies.

Examination on admission for cycle 3 was remarkable for oral candidiasis. Routine blood investigations identified potassium 2.7 mmol/l which was replaced with IV and oral potassium and albumin of 32 g/l. She developed nausea after day 2 and had one episode of vomiting.

On day 3, she was noted to be resting during the morning, however was easily rousable and able to stay awake. Chemotherapy was commenced at midday. Within 4 h, it was noted that she was difficult to rouse. Vital signs were within normal ranges. A blood gas confirmed a potassium of 2.7 mmol/l, but no other abnormality was identified and the pH was uncompensated and in the normal range. An ECG was unremarkable. It was hypothesized that she had developed ifosfamide encephalopathy and an EEG was obtained.

## Discussion

**Fig. 1:**
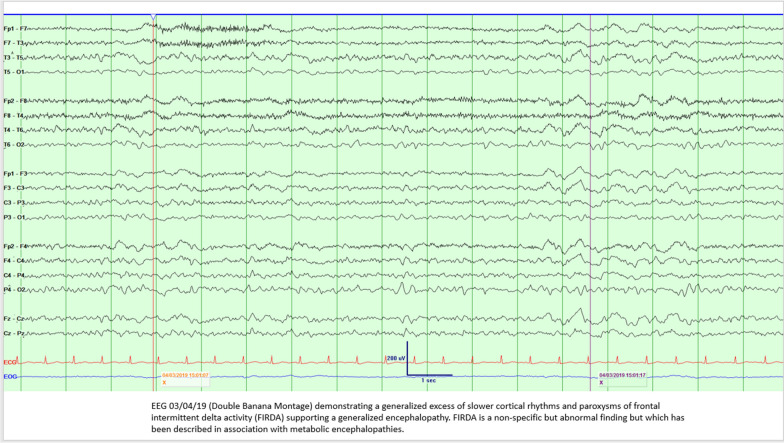
The EEG demonstrating FIRDA

In the EEG (Fig. 1) taken while the patient was encephalopathic, one can see the generalized features of periodic discharges, occasional triphasic morphology and intermittent delta activity with background attenuation. In addition to this, there is rhythmic delta activity seen intermittently. Unusually, this EEG demonstrates the unusual and rare finding of frontal intermittent rhythmic delta activity (FIRDA) [9]. FIRDA is a non-specific finding associated with frontal pathologies and has been descried in severe metabolic encephalopathies and in cases with significant mid-line shifts. This is a rarely seen phenomenon and was only identified in one case of a recent study by Gudson et al. [10].

Methylene blue, 50 mg intravenous (IV) slow push, was administered and there was a remarkable recovery with resolution of the drowsiness within 10 min of administration. On resolution of symptoms, the patient reported that she felt considerably better and that she was aware that she had been drowsy and unable to appropriately rouse herself. Oral thiamine was administered and the patient was admitted to the intensive care unit for monitoring. A further EEG was performed after resolution of symptoms, and all the previous features of encephalopathy were proven to have resolved.

Likely contributing factors to the ifosfamide-induced encephalopathy seen in this patient were her advanced age, low albumin, renal impairment manifesting in deranged electrolytes and the addition of aprepitant to her regimen.

## Conclusions

This case is interesting as it adds to evidence to benefit of methylene blue in the management of ifosfamide-induced encephalopathy, and also demonstrates the rarely observed EEG phenomenon of FIRDA during the ifosfamide-induced encephalopathy.

## Data Availability

Not applicable.

## Abbreviations

CT: Computed tomography
EEG: Electroencephalogram
FIRDA: Frontal rhythmic intermittent delta activity
IV: Intravenous
